# Evaluation of aliphatic acid metabolism in bladder cancer with the goal of guiding therapeutic treatment

**DOI:** 10.3389/fonc.2022.930038

**Published:** 2022-08-18

**Authors:** Tianbao Song, Kaixiang He, Jinzhuo Ning, Wei Li, Tao Xu, Weimin Yu, Ting Rao, Fan Cheng

**Affiliations:** ^1^ Department of Urology, Renmin Hospital of Wuhan University, Wuhan, China; ^2^ Department of Anesthesiology, Renmin Hospital of Wuhan University, Wuhan, China; ^3^ Department of Urology, Huanggang Central Hospital, Huanggang, China

**Keywords:** bladder cancer, aliphatic acid metabolism, immune therapy, prognosis biomarker, data mining bladder cancer

## Abstract

Urothelial bladder cancer (BLCA) is a common internal malignancy with a poor prognosis. The re-programming of lipid metabolism is necessary for cancer cell growth, proliferation, angiogenesis and invasion. However, the role of aliphatic acid metabolism genes in bladder cancer patients has not been explored. The samples’ gene expression and clinicopathological data were obtained from the Cancer Genome Atlas (TCGA) and the Gene Expression Omnibus (GEO). Univariate, multivariate, and LASSO Cox regression were used to develop a BLCA prognostic model. GSVA was used to assess function, whereas pRRophetic was used to assess chemotherapeutic drug sensitivity. The twelve-gene signature may define the tumor immune milieu, according to the risk score model. We compared the expression of aliphatic acid metabolism genes in malignant and non-cancerous tissues and chose 90 with a false discovery rate of 0.05 for The Cancer Genome Atlas cohort. The prognostic risk score model can effectively predict BLCA OS. A nomogram including age, clinical T stage, gender, grade, pathological stage, and clinical M stage was developed as an independent BLCA prognostic predictor. The halfmaximal inhibitory concentration (IC50) was used to assess chemotherapeutic medication response. Sorafenib and Pyrimethamine were used to treat patients with low risk scores more sensitively than patients with high risk scores. Immunotherapy candidates with CMS1 exhibited higher risk ratings. The aliphatic acid prognostic risk score model can assess metabolic trends. Clinical stage and molecular subtype may be used to categorize individuals using the risk score.With this new paradigm, future cancer treatment and immunotherapy may be tailored to the patient’s exact requirements.

## Introduction

Bladder cancer is the tenth most frequent kind of cancer globally, with an expected 549 393 new cases and 199 922 deaths from bladder cancer in 2018 (1). The majority of bladder cancers are urothelial carcinomas and are classified as per their therapeutic implications as non-muscle invasive bladder cancers (NMIBC) or muscle invasive bladder cancers (MIBC) (2). Tobacco use and occupational exposures (e.g. arylamines) are the primary risk factors for developing urothelial bladder cancer in the United States of America, and they also enhance the likelihood of recurrence (3). Transurethral resection of the bladder tumor (TURBT) is commonly advised in individuals with superficial bladder cancer, with or without intravesical therapy (3). The primarystay of therapy for muscle-invasive bladder cancer is radical cystectomy (RC) (4). The degree of bladder wall invasion is strongly linked with the clinical therapy of bladder cancers (5) Around 75% of individuals initially diagnosed with bladder cancer have NMIBC, with around 10% progressing to MIBC or metastatic bladder cancer (6). The expenditures of bladder cancer therapy, monitoring, and managing treatment-related side effects are significant (7). Despite advancements in early detection and systematic treatment of bladder cancer, some people continue to have recurrence or metastatic bladder cancer illness. As a result, it is critical to determine early diagnostic along with prognostic indicators of BLCA proliferation, as well as to develop novel ways for BLCA diagnosis and treatment, in order to optimize therapeutic results.

Fatty acids (FAs), a broad class of molecules made up of hydrocarbon chains with various lengths and degrees of desaturation, are the starting point for the synthesis of many lipids. FAs make up the hydrophobic tails of phospholipids, glycolipids, and cholesterol, which together make up a significant portion of biological membranes. In addition, second messengers that are produced in response to external stimuli, such as diacylglycerol (DAG) and phosphatidylinositol- 3,4,5-trisphosphate (PIP3), are also produced by membrane lipids. FAs may also be combined to form triacylglycerides (TAGs), nonpolar lipids that are produced and stored during periods of high nutritional availability and that, when broken down, release a significant amount of energy. In the majority of cancers, the tumor microenvironment is mostly composed of tumor cells and a variety of tumor stromal cells, cytokines and chemokines, immune cells, along with their mediators (8). Not only cytogenetics is involved in the tumor microenvironment, but also a knowledge of tumor activity in the surrounding milieu (9). Controlling tumor development and spread requires changing tumor cells and their microenvironment (10). The tumor microenvironment (TME) is pivotal for cancer prevention and immune suppression (11). Low oxygen levels in TME caused by an irregular tumor blood supply, high metabolic demand of the tumor, and even inflammation cause cells in the TME to switch to anaerobic metabolism (12). In the TME, tumor cells rely heavily on glucose metabolism for energy generation (13). For example, typical cells use glycolysis to convert the majority of glucose to pyruvate, and glycolysis in concert with oxidative phosphorylation in the mitochondria generates a large amount of energy. Nonetheless, since cancer cells catabolize glucose into lactate and produce inadequate energy, they need a greater glucose concentration for growth (14). The reprogramming of lipid metabolism in the TME is required for the formation of solid tumors (15). Recent research has shown a link between alterations in lipid metabolism and bladder cancer. Overexpression of aliphatic acid synthase (FASN) has been associated with a negative relationship between OS and recurrence. Additionally, blocking the AKT/mTOR signaling pathway resulted in a substantial reduction in BLCA cell proliferation and invasion when FASN expression was lowered. FASN may have a role in chemotherapeutic resistance development (16–21).

The genetic data from 414 BLCA samples were analyzed to provide a comprehensive knowledge of the aliphatic acid metabolic pattern and to create a prediction risk score model for aliphatic acids. The predictive risk score model accurately estimated the survival outcome of BLCA patients independently and effectively distinguished patients with BLCA who were resistant to several chemotherapeutic treatments. Furthermore, we investigated the relationship of the prognostic risk score model with the TME cell-infiltrating properties. The prognostic risk score model accurately classified BLCA patients as immunotherapy candidates, demonstrating that aliphatic acid metabolism is crucial for creating unique TME characterizations. These findings may provide fresh insight on the metabolic mechanism of BLCA and its treatment.

## Materials and procedures

### Data processing

The TCGA database’s raw RNA sequencing (RNA-seq) data patterns (https://www.cancer.gov/about-nci/organization/ccg/research/structural-genomics/tcga) were abstracted using the High Throughput Sequencing (HTSeq)-fragments per kilobase of transcript per million mapped reads (FPKM) workflow type, which included 414 BLCA and 19 normal bladder tissue samples. Additionally, we utilized the TCGA database to collect clinical data on 414 BLCA samples, consisting of gender, AJCC TNM stage, age, prognostic information along with pathological stage. GEO: Microarray data profiles for GSE13507 based on platform GPL6102 were abstracted from the GEO data resource (https://www.ncbi.nlm.nih.gov/geo/). We converted the Entrez Gene IDs of every sample into their respective gene symbols *via* the annotation platform. When several probes were used to target the same Entrez Gene ID, the mean value was utilized. Additionally, clinical data were gathered from the GEO database for each sample in GEO: GSE13507. Previously, 310 genes involved in aliphatic acid metabolism were identified. 310 common genes were picked from these genes in the TCGA, as well as GEO cohorts.

### DEG enrichment analyses in normal and cancerous tissue

The “limma” R package was adopted to discover differentially expressed genes involved in aliphatic acid metabolism between normal and malignant tissue samples. Genes having an FDR of 0.05 were regarded statistically significant. After that, the R package “org.Hs.eg.db” was adopted to transform the symbol gene for each DEG to an Entrez Gene ID. The study of GO and KEGG pathway enrichment on DEGs was performed using the R package “clusterProfiler” in order to determine the major biological features along with the cell function pathways. A difference that is statistically significant was regarded as having a p value (q value) of 0.05. Finally, we employed the R packages “enrichplot” and “ggplot2” to illustrate the results of the enrichment study.

### Devised and verified an approach for assessing prognostic risk

The training set included samples from the TCGA cohort, while the test set included samples from GEO: GSE13507. Using the samples’ unique identity, the expression levels of differentially expressed aliphatic acid metabolism-linked genes were first merged with the appropriate predicted outcomes. Genes associated with prognosis were identified from differentially expressed aliphatic acid metabolism-linked genes using univariate Cox regression assessment on the training set. We picked genes with a 0.05 p value. The “maftools” R package was adopted to determine gene mutations and correlations in BLCA samples from the training set. LASSO Cox regression was done to further analyze the genes related with prognosis in order to create a predictive risk score model for estimating OS in BLCA samples using the “glmnet” R package. A tenfold cross-verification was used to assess the model’s penalty parameter (λ). The approach outlined below was adopted to determine the risk score for every sample. $\text{Risk Score}=\sum_{1}^{i}\left(Coefi*ExpGenei\right)$ . The “Coef” column includes non-zero regression coefficients determined *via* the LASSO Cox approach, and the “ExpGene” column provides the expression levels of genes included in the predictive risk score model. To classify all samples into low-, as well as high-risk score categories, the median value of risk scores was employed. Kaplan-Meier approach along with the log-rank test was adopted to compare the OS of low-risk and high-risk score groups. To assess the predictive accuracy of the predictive risk score model, a time-dependent receiver operating characteristic (ROC) curve was generated using the R package “survivalROC”. Finally, the test set was adopted to examine the prognostic risk score model’s reliability and applicability.

### Principal component analysis (PCA) was compared before to and during the construction of a prognostic risk score

The limma R package was adopted to perform PCA on gene expression profiles before to and after the training set’s predictive risk score model in order to grasp the significant difference between low and high-risk score groups. PCA was first used to analyze the expression patterns of all differentially expressed genes linked with aliphatic acid metabolism. PCA was then employed to assess the expression patterns of genes in the predictive risk score model. Finally, utilizing two-dimensional diagrams structured around the first two fundamental components, the ggplot2 tool was utilized to illustrate the PCA results.

### Comparative analysis of risk scores and clinical features

Using the R tool “CMScaller,” all samples in the TCGA cohort were categorised into CMSs on the basis of their features. The risk score for every sample was merged with the related clinical features using the sample ID. The limma R software was used to examine the relationship of the risk scores with clinical data, consisting of gender, pathological stages, CMS, age, as well as AJCC TNM stages. Additionally, the TCGA database was utilized to ascertain the level of immunological checkpoint expression (PD-1, PD-L1, and CTLA4). The levels of expression of immunological checkpoints were then compared between groups with low and high-risk scores. Clinical data on CRC from the GEO cohort were evaluated to determine the relationship between risk scores and clinical features. This included the presence of TP53, TTN, KMT2D, MUC16, ARID1A and KDM6A mutations. To explore disparities in risk scores across samples, they were separated into two groups on the basis of their clinical features. The Wilcoxon rank-sum along with the Kruskal-Wallis (K-W) tests were utilized to compare two groups and more than two groups. A p value of 0.05 was judged statistically significant.

### GSVA

The “GSVA” R tool was adopted to compare biological processes between low- and high-risk score groups. GSVA is a non-parametric along with unsupervised technique for analyzing changes in biological pathways or processes using an expression matrix sample. The reference gene sets were generated from the molecular signatures data resource’s “c2.cp.kegg.v7.1.symbols” gene sets (https://www.gsea-msigdb.org/gsea/msigdb). FDR0.05 signified a statistically remarkable enrichment cascade. The IC50 of 5-FU was estimated in each sample using a typical comparison of the low- with the high-risk score groups using the pRRophetic R program. The IC50 number indicates a substance’s capacity to inhibit certain biological or metabolic processes. To determine the extent of immune-linked invasion in every sample from the TCGA cohort, ssGSEA was carried out in the R packages “GSEABase” along with “GSVA”. The gene sets were compiled in order to evaluate immune-linked aspects in TME from prior research, and they covered a diverse range of human immune cell sub-types, as well as immune-linked behaviors, for instance CD8+ T cell, B cell, and T cell co-stimulation (**Table S1**). The ssGSEA method calculated enrichment scores based on the relative frequency of expression of each immune-linked characteristic in every sample. The enrichment scores of people with low- and high-risk scores were compared. Additionally, the link between genes associated with prognosis and immune cells was investigated. A p value of 0.05 signified statistically significant.

### PPI network

We began by examining the RNA-seq data patterns of low- along with high-risk score groups using the limma R tool. DEGs were classified as genes having a 0.05 corrected p-value. The DEGs were evaluated using the STRING online data resource (version 11.0; https://string-db.org/), which yielded PPI network data with a median confidence level of more than 0.40. (**Table S2**). Following that, Cytoscape was utilized to do further analysis and visualization of the PPI network data (v3.7.2). The Cytoscape plugin cytoHubba (v0.1) was adopted to search for hub genes across all DEGs using topological approaches. Following that, the genes that were differently expressed between normal and BLCA colorectum tissue were discovered. The R clusterProfiler resource was adopted to analyze the genes for GO and KEGG enrichment. Finally, on the bsais of the median expression level of the hub genes, all samples were stratified into low-, as well as high-expression groups. To assess whether there was a difference in survival between the two groups, Kaplan-Meier assessment was utilized. Immune cell invasion into hub genes linked to prognosis were compared.

### A nomogram has been designed for the purpose of estimating OS

A nomogram for BLCA OS prediction was created using the R package “rms” with the TCGA cohort. It included age, pathological stage, gender, as well as predictive risk score. Using time-dependent calibration curves, the nomogram’s accuracy was projected. Additionally, multivariate Cox regression assessment was adopted to investigate if the predictive risk score model could be used as an independent predictor of OS in colorectal cancer patients. The AUC was then determined *via* the online ROC curves to determine the nomogram’s prognostic value.

### HPA dataset

The Human Protein Atlas (HPA), a data resource that offers immunohistochemistry-based expression data for around 20 most prevalent kinds of malignancies, 12 individual tumors in each cancer type, was utilized to assess the protein contents of the 5 hub genes in human healthy and BLCA tissues (22).

### Conducts statistical analysis

The two groups were compared *via* the Wilcoxon rank-sum test. Three or more groups were compared *via* the K-W test. The survival differences between the low- and high-risk score groups were examined through the Kaplan-Meier approach. The independent determinants of OS in BLCA were identified through multivariate Cox regression. To determine the predictive ability of the prognostic risk score model and nomogram, receiver operating characteristic curves were performed. All the statistical analyses were implemented in R v4.0.0, with p<0.05 signifying statistical significance.

## Results

### Analysis of normal and malignant tissue samples for enrichment

**Figure 1:**
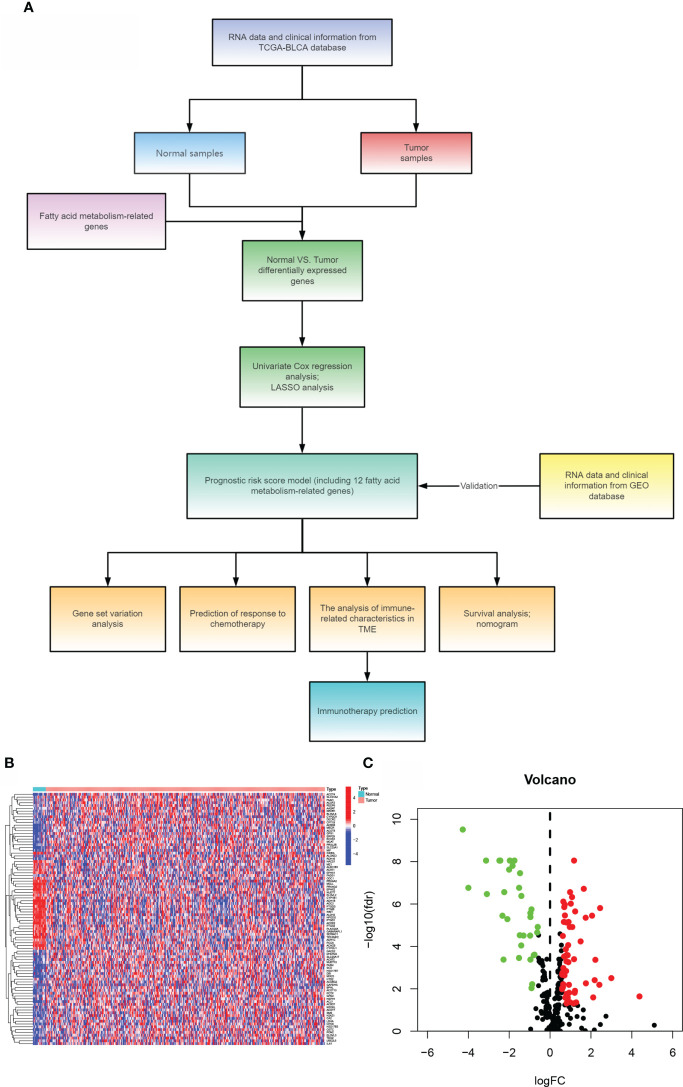
Comparing bladder cancer with non-tumorous tissue samples from the TCGA cohort. **(A)**The article’s flow chart. **(B)** A heat map of 90 differentially expressed genes involved in aliphatic acid metabolism. **(C)** The volcano map of 90 differentially expressed genes involved in aliphatic acid metabolism.

The article’s flow chart is given in **Figure 1A**. We compared the expression levels of genes participating in aliphatic acid metabolism in non-tumorous and cancer tissue samples, and 90 genes with an FDR 0.05 were chosen for inclusion in the TCGA cohort. In cancer tissue samples, 58 genes were elevated and 32 were downregulated. The differentially expressed genes are given in **Figures 1B, C**. (DEGs). The DEGs were then analyzed for Gene Ontology (GO) enrichment. Among the biological processes, aliphatic acid metabolism, organic acid biosynthetic, carboxylic acid biosynthetic, and aliphatic acid derivative metabolic process were substantially enriched GO keywords (**Figure S1A**). The findings of an enrichment study using the KEGG revealed that aliphatic acid metabolism, aliphatic acid degradation, metabolism of arachidonic acid, tryptophan metabolism, and were all significantly enriched KEGG keywords (**Figure S1B**). These findings indicate that aliphatic acid metabolism harbors an indispensable role in the development of BLCA.

### Development of a prognostic risk score model using the training set

**Figure 2:**
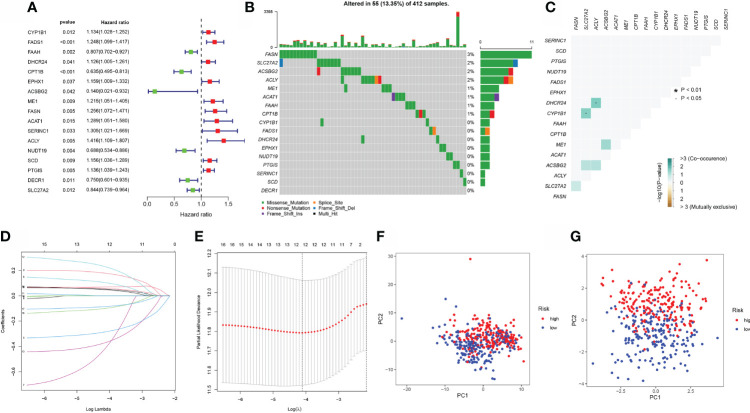
Model development for prognostic risk assessment **(A)** Forrest plot of 17 genes involved in aliphatic acid metabolism associated with prognosis. **(B)** Mutation frequency of 17 genes involved in aliphatic acid metabolism in 412 individuals with BLCA from the TCGA cohort. **(C)** Co-occurrence along with exclusion analysis of mutations in 17 genes involved in aliphatic acid metabolism. Green indicates co-occurrence; purple indicates exclusion. **(D)** LASSO coefficients for the 17 genes involved in aliphatic acid metabolism. **(E)** Gene discovery with the purpose of developing a predictive risk score model. **(F)** Principal component analysis of all BLCA genes involved in aliphatic acid metabolism. **(G)** Principal component analysis using a risk score for aliphatic acid metabolism to differentiate cancers from non-malignant tissue samples in the TCGA cohort. The green group designated high-risk patients, whereas the red group designated low-risk individuals. *p < 0.05.

The training set was constructed using TCGA cohort samples. Univariate Cox regression was used to assess 90 differentially expressed aliphatic acid metabolism genes. A total of 17 genes involved with prognosis were identified with a p value of 0.05. (**Figure 2A** and **Table 1**). We began by summarizing the somatic mutation profile of 17 genes involved in aliphatic acid metabolism related with poor prognosis. As illustrated in **Figure 2B**, a total of 55 of 412 BLCA samples had mutations in genes involved in aliphatic acid metabolism, representing a 13.55% frequency. FASN was mutated more frequently than SLC27A2. DECR1, on the other hand, did not exhibit any changes in BLCA samples. Additional analyses established a correlation between the CYP1B1 and SLC27A2 mutations, as well as the DHCR24 and ACLY mutations (**Figure 2C**). The number of genes was then reduced using Cox regression analysis with the least absolute shrinkage and selection operator (LASSO). Finally, twelve genes (CYP1B1, FADS1, CPT1B, EPHX1, ACSBG2, FASN, ACLY, NUDT19, SCD, PTGIS, DECR1, and SLC27A2) were used to develop a predictive risk score model (CYP1B1, FADS1, CPT1B, EPHX1, ACSBG2, FASN, ACLY, NUDT19, SCD, PTGIS, DECR1, and SLC27A2) (**Figures 2D, E**). The risk score for each sample was calculated as follows: Risk score = (0.051627765239571) * CYP1B1+(0.069487777237826) * FADS1+(-0.284173428370957) * CPT1B+(0.102797313048634) * EPHX1+(-0.40870133580594) * ACSBG2+(0.177530542982211) * FASN+(0.228151304346159) * ACSL6. The risk score model was utilized to fully identify low- and high-risk BLCA samples (**Figures 2F, G**).

**Table 1:** The unicox results of aliphatic acid metabolism related genes in BLCA.

id	HR	HR.95L	HR.95H	pvalue
CYP1B1	1.134194	1.027638	1.2518	0.012365
FADS1	1.247846	1.098894	1.416989	0.00064
FAAH	0.80687	0.702405	0.926871	0.002418
DHCR24	1.125808	1.004933	1.261222	0.040866
CPT1B	0.634687	0.495423	0.8131	0.000322
EPHX1	1.159196	1.00884	1.331962	0.037149
ACSBG2	0.140267	0.021113	0.931864	0.042052
ME1	1.214928	1.050692	1.404837	0.008607
FASN	1.255983	1.072129	1.471366	0.004766
ACAT1	1.288816	1.050969	1.580492	0.014787
SERINC1	1.305306	1.021072	1.668663	0.033471
ACLY	1.415876	1.109378	1.807053	0.005207
NUDT19	0.688117	0.534144	0.886474	0.003823
SCD	1.155528	1.036247	1.28854	0.00931
PTGIS	1.136026	1.038623	1.242563	0.005294
DECR1	0.749596	0.600636	0.935498	0.010778
SLC27A2	0.843864	0.738861	0.963789	0.012281

### The correlation between the risk score and clinical characteristics

**Figure 3:**
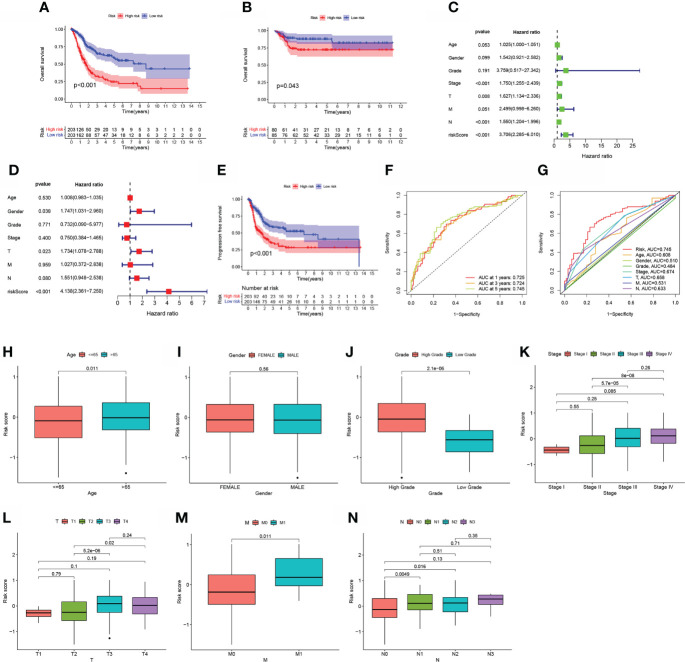
The estimation efficacy of the aliphatic acid metabolism score model in predicting BLCA patients’ survival status. **(A, B)** Comparison of OS in the training and test sets between low- BLCA and high- BLCA risk score groups. **(C, D)** The forest plot depicts the data of the TCGA data set univariate along with multivariate Cox regression.**(E)** Comparison of progress-free survival (PFS) in the TCGA cohort between low- BLCA and high-BLCA risk score groups. **(F)** The estimation potential of the risk score as determined by ROC curves in the 1,3,5-year TCGA cohort. The AUC values are 0.725, 0.724, and 0.745, respectively. **(G)** Receiver operating characteristic curves for the TCGA cohort’s aliphatic acid metabolism score along with clinico-pathological features. **(H–N)** The relationship of the risk score with clinicopathological characteristics, such as age **(H)**, gender **(I)**, grade **(J)**, clinical stage **(K)**, clinical T stage **(L)**, clinical N stage **(M)**, and clinical M stage **(N)**.

The cutoff value was chosen at the median of the risk assessments in the training set. On the basis of the threshold value above, the sample risk scores were sorted and categorized into low (n = 203) and high (n = 203) risk score categories. In the TCGA, samples with a high-BLCA risk score had a worse prognosis than samples with a low risk score (p<0.001; **Figure 3A**). Using the training set’s threshold value, test group samples from GEO: GSE13507 were divided into low-BLCA (n = 85) and high -BLCA (n = 80) risk score groups to verify the predictive risk score model. The high-BLCA risk group’s samples had a poorer prognosis than the low-BLCA risk group’s samples (p=0.043; **Figure 3B**), indicating that the prognostic risk score model may reliably predict OS in BLCA. Only the risk score and clinical T stage were independent predictors of OS in multivariate analysis among the criteria linked to OS in univariate analysis, which included lymph node status, clinical stage, T stage, and risk score (**Figures 3C, D**). There was a strong link between PFS (progression-free survival) and risk score in the TCGA-BLCA cohort (p0.001; **Figure 3E**). To verify the predictive risk score model’s accuracy, a time-dependent ROC curve was drawn at 1, 3, and 5 years (**Figure 3F**). The AUC indicated that the risk score (AUC = 0.745) was more predictive of survival than age (AUC = 0.608), pathological stage (AUC = 0.674), clinical T stage (AUC = 0.658), and clinical N stage (AUC = 0.633; **Figure 3G**). We investigated the distribution of risk scores in matched samples by age, gender, grade, pathological stage, and AJCC TNM Classification of Malignant Tumors (TNM) stage. Although there were no significant associations between risk scores and gender, advanced pathological stages, or AJCC-T (tumor invasion) (**Figures 3I, K, L**), higher risk scores were associated with increased age (p=0.011; **Figure 3H**), grade (p<0.001; **Figure 3J**), AJCC-M (distal metastasis) (p = 0.011; **Figure 3M**), and AJCC-N (lymphoid metastasis) (**Figure 3N**).

### The creation of a nomogram for survival prediction

**Figure 4:**
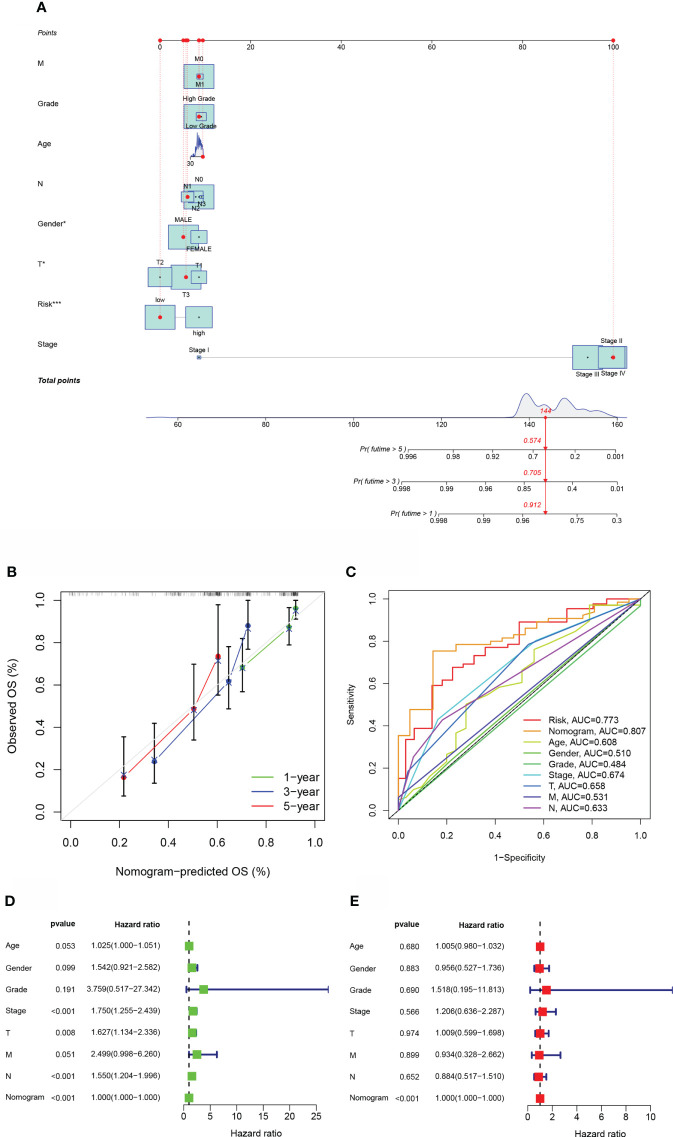
The estimation potential of the aliphatic acid metabolism score in conjunction with clinicopathological features in patients from the TCGA cohort with regard to overall survival. **(A)** Nomogram for estimating OS in TCGA cohort participants. **(B)** The nomogram’s calibration plots. The x axis depicts expected survival, whereas the y axis designates the actual survival. **(C)** Receiver operating characteristic curves for the TCGA cohort’s aliphatic acid metabolism score, clinical pathological features, and nomogram. **(D, E)** Analysis of the nomogram using univariate coupled with multivariate Cox regression. *p < 0.05, ***p < 0.001.

For OS prediction in BLCA samples, a nomogram with integrated age, grade, pathological stage, clinical T stage, clinical M stage, gender, and clinical N stage, as well as a predictive risk score model, was built (**Figure 4A**). Calibration curves at one-year, three-years, as well as five-years exhibited that the nomogram successfully estimated the OS of CRC patients, as seen in **Figures 4B**. The AUC indicated that the nomogram (AUC = 0.807) was more predictive of survival than a single indication, such as risk (AUC = 0.773) or clinical stage (AUC = 0.674) (**Figure 4C**). Cox regression study, both univariate and multivariate, demonstrated that Nomogram is an independent prognostic predictor of BLCA (**Figures 4D, E**).

### Analyses of gene set variation (GSVA)

**Figure 5:**
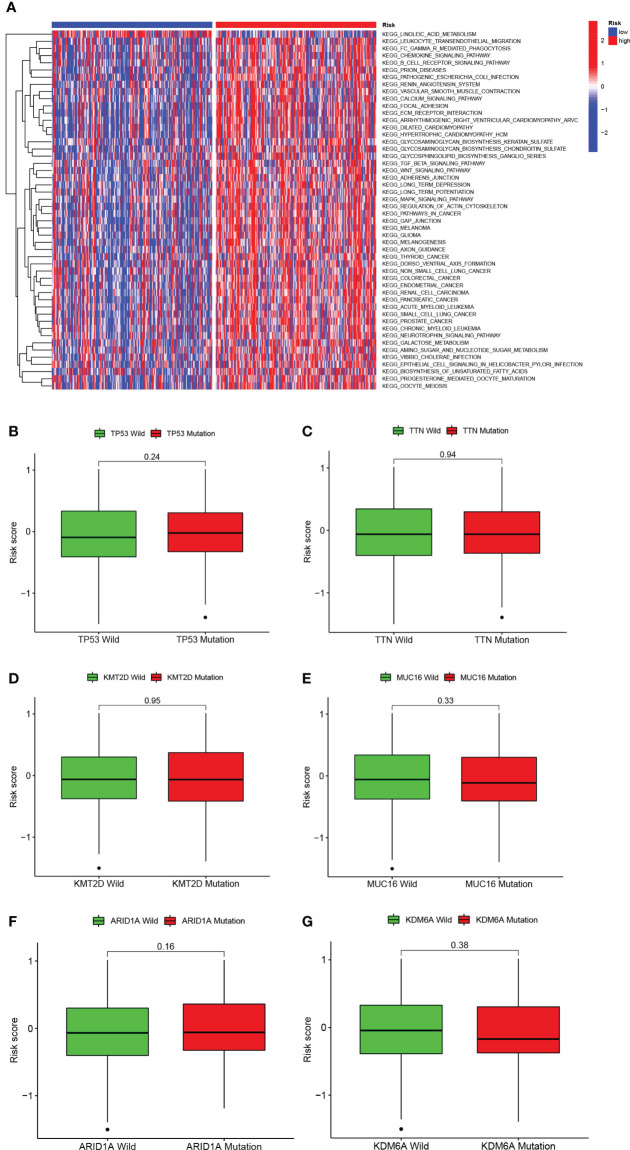
GSVA and mutation analysis of a model of aliphatic acid metabolism. **(A)** Heatmap depicting the enrichment of GSVA in low- BLCA and high- BLCA risk score categories. **(B–G)** Differences in lipid metabolism score between several kinds of gene mutations, including TP53 mutation **(B)**, TTN mutation **(C)**, KMT2D status **(D)**, MUC16 status **(E)**, ARID1A status **(F)**, and KDM6A status **(B, G)**.

To investigate the biological behaviors of the two groups, GSVA enrichment was performed *via* the gene sets “c2.cp.kegg.v7.2” retrieved from the Molecular Signatures Database (MSigDB). The high-risk score enriched the majority of metabolic pathways, including aliphatic acid metabolism, and the majority of signaling pathways (**Figure 5A**). Additionally, individuals with TP53, TTN, KMT2D, MUC16, ARID1A, or KDM6A mutations had a nonsignificant risk value compared to those without the mutation. (See **Figures 5B–G**).

### Chemotherapy reaction

**Figure 6:**
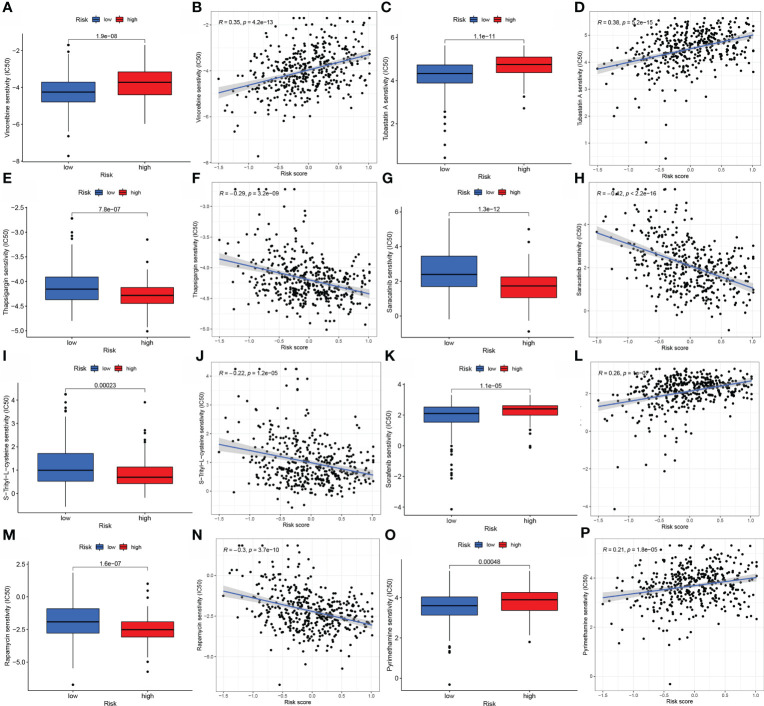
A model of aliphatic acid metabolism in the context of chemotherapy. The relationship between patient risk scores and predicted IC50 values, as well as the response variations between the low- BLCA and the high- BLCA risk score groups for several chemotherapeutic agents. **(A–D)** Vinorelbine, **(E, F)** Thapsigargin **(G, H)** Saracatinib, S-Trityl-L-cysteine **(I, J)**, Sorafenib **(K, L)**, Rapamycin **(M, N)**, and Pyrimethamine **(O, P)**.

Given the association of the risk score with dismal prognosis, the link of the risk score with chemoresistance was investigated. The halfmaximal inhibitory concentration (IC50) was used to estimate treatment response to several chemotherapeutic drugs in the TCGA cohort using the R package “pRRophetic”. Low-risk score samples were more susceptible to Vinorelbine, Tubastatin A, Sorafenib, and Pyrimethamine, which are more generally recommended for advanced BLCA treatment. Thapsigargin, Sunitinib, S-Trityl-L-cysteine, and Rapamycin were more sensitive to high-risk score samples, which were more generally recommended for treating terminal BLCA (**Figures 6A–P**).

### Characteristics of the immune system in the low- BLCA and high- BLCA risk score groups

**Figure 7:**
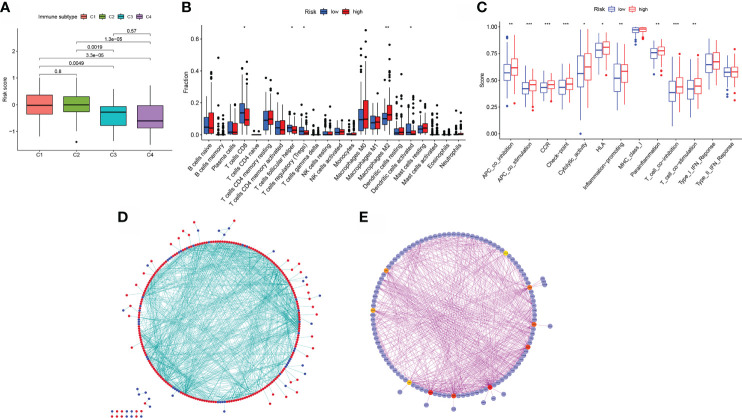
Model of aliphatic acid metabolism in relation to immunotherapy. **(A)** The difference in risk scores between CMS subtypes. **(B)** The difference in immune infiltration between high- BLCA risk and low- BLCA risk scores. **(C)** The difference in the known function linked with immune modulation between subjects with a high- BLCA risk score and those with a low- BLCA risk score. **(D)** Cytoscape-processed PPI network (red): DEGs with a high level of expression in the high- BLCA risk score group; blue: DEGs with a low level of expression in the low-BLCA risk score group. **(E)** cytoHubba’s top ten hub genes. *p < 0.05, **p < 0.01, ***p < 0.001.

Additionally, individuals with the CMS1 phenotype who were immunotherapy candidates had higher BLCA risk scores (**Figure 7A**), demonstrating that quantifying the aliphatic acid metabolism risk score is an unique and robust biomarker for assessing prognosis along with clinical responsiveness to immunotherapy. The group was extremely densely infiltrated with immune-suppressive cells, including CD8 T cells, T cell follicular helper cells, T cell regulatory cells (Tregs), M2 macrophages, and activated dendritic cells, corresponding with the high- BLCA risk group’s survival disadvantage (**Figure 7B**). Additionally, para-inflammatory, APC co-inhibition, HLA, APC co-stimulation, CCR, check-point cytolytic activity, pro-inflammatory, T cell co-inhibition, along with T cell co-stimulation were activated in the high-risk group, showing that individuals with suppressed immunity may react to immunotherapy (**Figure 7C**).

### Network of DEGs with protein-protein interactions (PPIs) in low- BLCA and high- BLCA risk score groups

**Figure 8:**
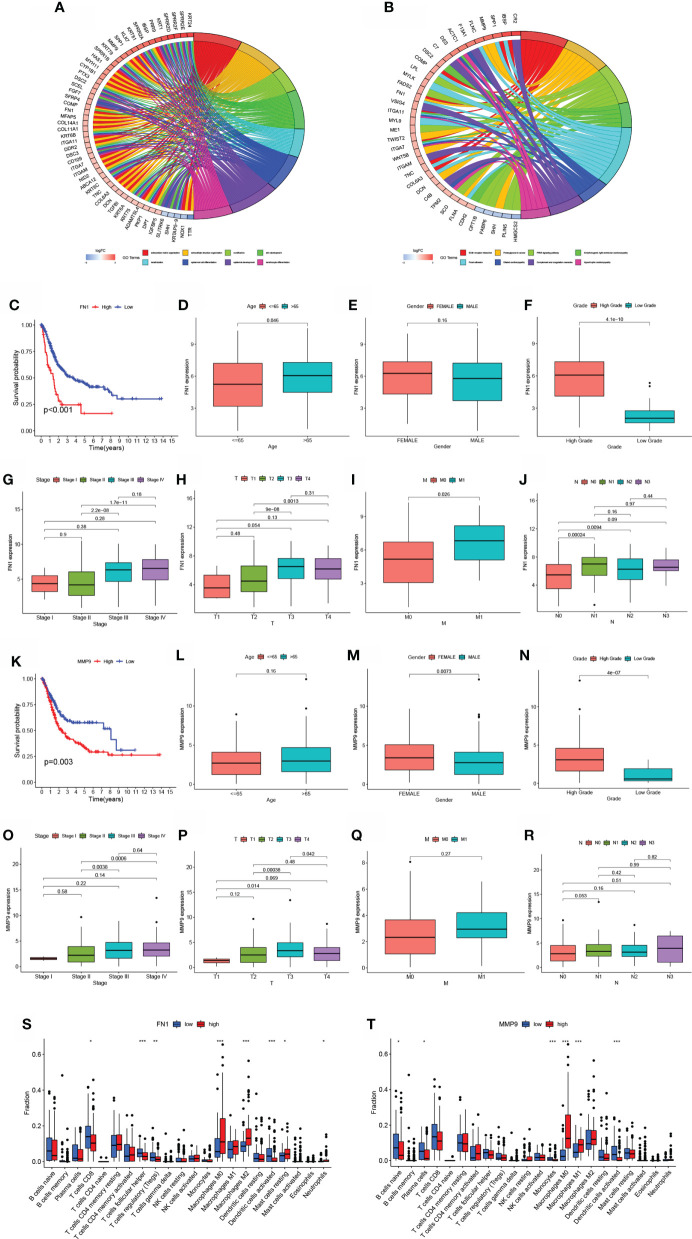
Hub gene prognosis and immunological analysis. **(A, B)** GO along with KEGG enrichment analysis findings for hub genes. **(E)** Survival study of patients classified into subgroups based on FN1 mRNA expression. **(D–J)** The difference in FN1 mRNA expression between patients with various clinical features. **(K)** Survival analysis for patients classified into subgroups based on MMP9 mRNA expression. **(L–R)** The difference in MMP9 mRNA expression between distinct clinical features. **(S–T)** The number of cells invading the TME in patients with varying levels of FN1 and MMP9 mRNA expression. *p < 0.05, **p < 0.01, ***p < 0.001.

**Figure 9:**
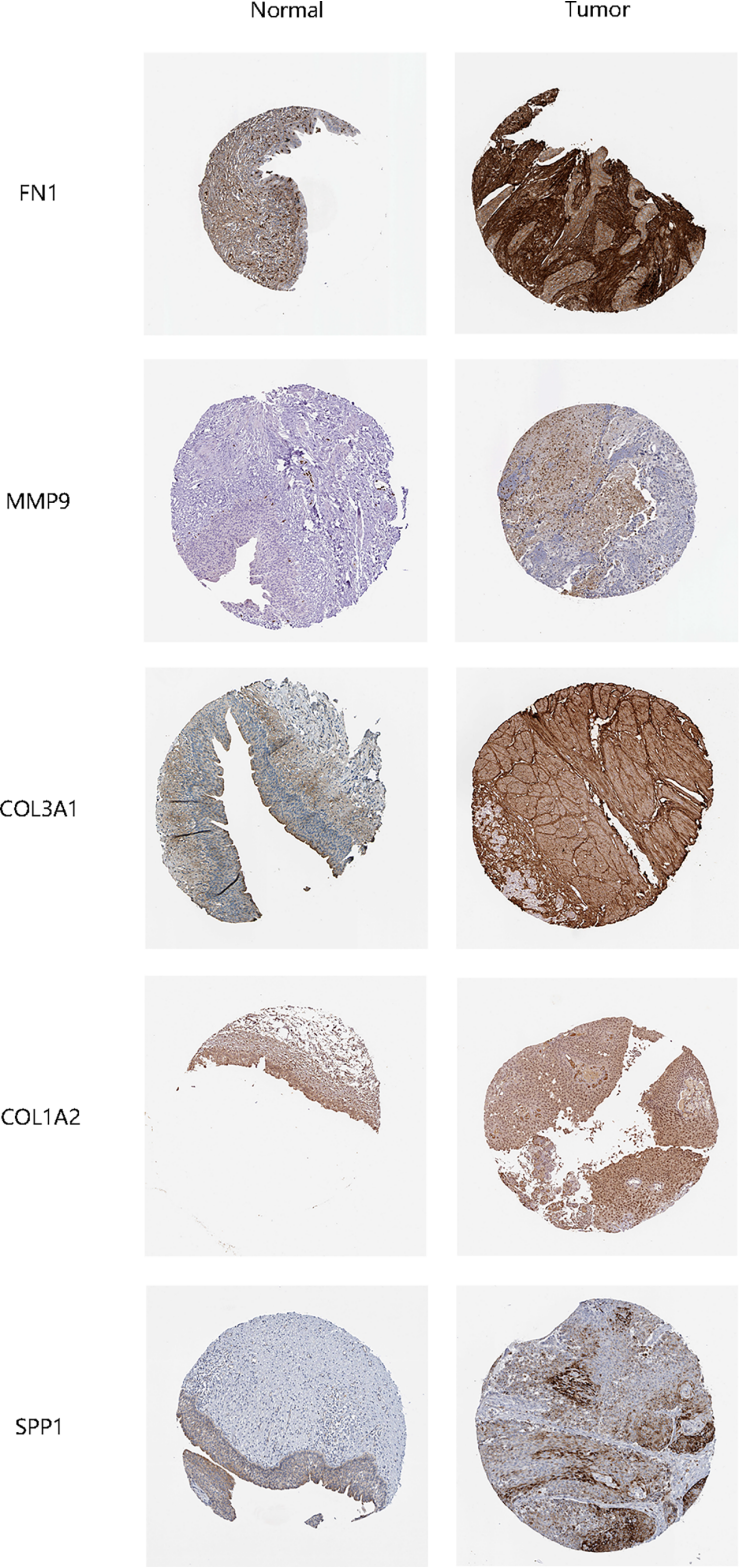
Validation of hub gene protein expression levels.

The STRING online data resource was utilized to examine the DEGs’ expression patterns in low-BLCA and high- BLCA risk groups. **Figure S2A** illustrates how the PPI network was created utilizing the DEGs. The PPI network data were generated and visualized through the Cytoscape program. DEG interaction is given in **Figure 7D**, with upregulated genes in the high- BLCA risk score group highlighted in red and downregulated genes in the low-BLCA risk score group highlighted in blue. Cytoscape’s cytoHubba plug-in was adopted to deduce the hub genes from the DEGs. As seen in **Figure 7E**, a total of ten genes were chosen from the network. The degree approach was used to rank FN1, COL1A2, MMP9, COL3A1, SPP1, ITGAM, DCN, ACTA2, LOX, and CXCL12. The changes in gene expression between healthy and malignant tissues were then compared. To get a better understanding of the function of eight distinct hub genes, we carried out GO along with KEGG analyses using the R package “GOplot.” The genes were shown to be participate in organization of the extracellular matrix, cornification, extracellular structure organization, skin development, keratinization, epidermal cell differentiation, epidermis development, and keratinocyte differentiation (**Figure 8A**). According to KEGG, these genes were enriched in the following categories: Focal adhesion, ECM receptor cross talk, Proteoglycans in cancer, PPAR signaling cascade, Dilated cardiomyopathy, Arrhythmogenic right ventricular cardiomyopathy, Complement and coagulation cascades, and Hypertrophic cardiomyopathy (**Figure 8B**). We examine the top five HUB genes in this section. The survival study revealed a significant association between FN1, MMP9, COL1A2, COL3A1, and SPP1 mRNA expression of the hub genes and the prognosis of BLCA patients (**Figures 8C, K**; **Figures S2A, S2C, S2E**). Additionally, expression levels rise with increasing age, grade, clinical M stage, and clinical N stage (**Figures 8D–J**). The expression of FN1 was shown to be related with a bad prognosis. The difference in TME immune cell invasion between patients with high and low FN1 expression was investigated using the FN1 median expression potential as a threshold value. Tumors expressing high levels of FN1 demonstrated considerably greater infiltration of M2 macrophages, activated dendritic cells, follicular T cells, and M0 macrophages, than tumors expressing low levels of FN1 (**Figure 8S**). Also, the expression of MM9, COL1A2, COL3A1 and SPP1 was shown to be related with a bad prognosis (**Figures 8K–R**; **Figure S3**). The results of immune infiltration of MMP9, COL1A2, COL3A1 and SPP1 were showed in **Figure 8T**; **Figures S2B, S2D, S2F**. Additionally, we used the HPA database to explore the protein levels of these three genes. The findings indicated that BLCA tissues included significantly greater levels of FN1, MMP9, COL1A2, COL3A1, and SPP1 (**Figures 9**).

## Discussion

One of the hallmarks of cancer is metabolic re-programming, and each metabolic state has a unique molecular signature that reflects a varied prognosis (23). Alterations in food metabolism in tumor stroma are being recognized as a critical component of cancer-linked metabolic reprogramming (24). It is commonly accepted that several oncogenic mechanisms may trigger glucose metabolic reprogramming (25). The Myc protein acts as a critical regulator of metabolism, participating in metabolic re-programming processes for instance glucose and glutamine metabolic re-programming, as well as serine synthesis, all of which contribute to cancer cell proliferation (26). Over the past two decades, remarkable advancements have been achieved in diagnosing and treating BLCA. While the majority of bladder cancers are non-muscle invasive upon diagnosis, the high relapse rate and risk of progression to invasive disease need periodic surveillance cystoscopy, resulting to bladder cancer being one of the most costly types of cancer to treat (27). Relapse of bladder cancer, emergence of resistance to drugs, along with a high rate of disease progression are significant obstacles to bladder cancer treatment, underscoring the fundamental need for the identification of novel biomarkers in the clinical diagnosis along with treatment of bladder cancer (28). This is the first research to investigate the link between genes involved in aliphatic acid metabolism and BLCA. Cox regression with univariate LASSO and Cox regression with univariate Cox Using 90 differentially expressed aliphatic acid metabolism-linked genes from TCGA and GEO tumor and normal BLCA tissue samples, we created a predictive risk score model. To get a better comprehension of the involvement of these genes in BLCA, we employed a predictive risk score model to estimate OS in the training set of BLCA patients. There were disparities in survival between patients with a low-risk BLCA score and those with a high-risk BLCA score. In the test set, the same result was obtained, exhibiting that the predictive risk score model is capable of identifying individuals at risk of poor survival. In multivariable analysis, the predictive risk score model was revealed as the independent predictive factor. Additionally, the predictive ability of the predictive risk score model was enhanced by including a few chosen clinicopathological characteristics into a risk-assessment nomogram. To get a better understanding of the predictive risk score model’s involvement in BLCA, we compared the responsiveness of patients with low- and high-risk ratings to pharmacological therapy. As previously reported, the risk score was favorably related with chemoresistance to various chemotherapeutic treatments. Patients harboring higher risk scores in the BLCA group had a shorter PFS, showing that the aliphatic acid predictive risk score model may be used to tailor BLCA therapy to individual patients. Patients harboring a high-risk score had a strong activation state of the stroma, indicating chemoresistance. It was thought that individuals with a high-risk score would not benefit from immunotherapy due to the development of chemoresistance. As a result, it is critical to identify immunotherapy candidates in clinical practice. Patients with a high-risk score had a higher concentration of inhibitory immune cells, such as Tregs and others, as well as immune-inflamed cells. Additionally, individuals with a high-risk score had activation of check-point cytolytic activity, PC co-inhibition, CCR, HLA, pro-inflammatory, para-inflammatory, APC co-stimulation, T cell co-inhibition, and T cell co-stimulation, suggesting that they are immunotherapy candidates. Due to the significant disparities in risk scores between the low-risk and high-risk groups, the distinct genes in the two groups were further investigated.

FN1 is a glycoprotein present in the extracellular matrix, as well as on the cell surface that stimulates cell adhesion along with migration, both of which are important in the onset and progress of cancer (29). In oral squamous cell carcinoma, upregulated FN1 was linked to a dismal prognosis and resulted in lymphangiogenesis, along with lymph node metastases (30). Overexpression of the FN1 gene has been implicated as a significant predictor of thyroid cancer aggressiveness and has also been shown in gastrointestinal carcinoma, renal carcinoma, hepatocellular carcinoma, and head/neck cancer (30, 31). MMP9 is a matrix metalloproteinase that aids cancer infiltration, metastasis, as well as angiogenesis (32). In tongue squamous cell carcinoma, MMP9 expression is elevated in neoplastic cells along the infiltration front, and MMP9 expression in histologically negative surgical margins of oral squamous cell carcinoma (OSCC) is a predictor of tumor recurrence (33). MMP9 overexpression is required for the advancement of a variety of tumor kinds, consisting of esophageal squamous cell carcinoma, bladder cancer, and intrahepatic cholangiocarcinoma (34). COL3A1, the extracellular matrix gene, which was first identified as a cause of autosomal dominant Ehlers-Danlos disease, was subsequently reported to be dramatically changed in individuals with melanoma (35). COL3A1 expression is related with a worse OS rate in patients with epithelial ovarian cancer in four primary tissue types (36). COL3A1 has been found to be over-expressed in a range of cancers, such as bladder cancer, glioblastoma, and gastric cancer, in addition to its normal expression in connective tissues (37). Collagen factors COL1A1, COL1A2, and COL3A1 were often implicated in carcinogenesis or metastasis in a variety of tumor types, for instance breast cancer, gastric cancer, and cervical cancer (38). The molecules controlled by COL1A2 are also related with cellular behaviors, as well as signaling cascades in CRC, which may be a significant factor in the high rate of relapse and dismal prognosis seen in individuals with CRC with reduced COL1A2 expression (39). SPP1 is over-expressed in a variety of malignant neoplasms, consisting of medullary thyroid cancer, HCC, as well as colorectal cancer, and is involved in metastasis and tumorigenesis (40). SPP1 acts as a CD44 ligand in glioma, promoting the production of the oncogene EPAS-1 and aggresive glioma development. SPP1 expression is related with poor survival in colorectal cancer patients with positive venous invasion and advanced TNM stage (41). Further research, however, will be necessary to determine the therapeutic use of these five hub genes in human bladder cancer. In this study, transcriptome profiling associated with aliphatic acid metabolism was carefully examined, and a risk-predictive signature based on survival and genes associated with aliphatic acid metabolism in BLCA patients was developed. However, there are still a few limitations that should be taken into account when interpreting the findings. The included genes were identified using information about their involvement in the illness’ development that was already known, but prospective data are needed to prove their clinical importance. In order to determine whether the signature has any potential therapeutic applications, independent external validation is still required. The signature was produced and confirmed using retrospective datasets that were made accessible to the public.

## Conclusions

The aliphatic acid predictive risk score model may be utilized to decipher the patterns of aliphatic acid metabolism. Individuals may be classified using the risk score based on their clinicopathological characteristics, such as clinical stage and molecular subtype. Additionally, the risk score is related to patient prognosis and may be used to forecast therapy sensitivity. As a result, clinical practice may be more effectively guided by risk score and clinical stage, resulting in a more tailored approach to clinical follow-up. These results provide a unique, efficient, as well as accurate prognosis and response prediction model for chemotherapy and immunotherapy, paving the path for future tailored cancer chemotherapy and immunotherapy.

## Data availability statement

The original contributions presented in the study are included in the article/**Supplementary Material**. Further inquiries can be directed to the corresponding authors.

## Author contributions

TS, KH, and JN coordinated to collect, as well as analyze data, and discussed the details, and wrote this article. Considering that authors TS, KH, and JN contributed equally to the work, they should be considered co-first authors. WL, TX, and WY analyzed and typed setting the data, figures and tables. FC and TR analyzed the data and modified this manuscript together and were primary contributors in writing the manuscript. FC and TR should be regarded as co-corresponding authors. Each person was assigned specific responsibilities, resulting in a clear division of labor. The final text was reviewed and approved by all writers.

## Funding

This research was supported by funding from the Chinese National Natural Science Foundation (no. 81800617 and 81870471) and the Hubei Province’s Science and Technology Major Project (2019AEA170).

## Acknowledgments

We would like to acknowledge all of the subjects for their support with data.

## Conflict of interest

The authors declare that the research was conducted in the absence of any commercial or financial relationships that could be construed as a potential conflict of interest.

## Publisher’s note

All claims expressed in this article are solely those of the authors and do not necessarily represent those of their affiliated organizations, or those of the publisher, the editors and the reviewers. Any product that may be evaluated in this article, or claim that may be made by its manufacturer, is not guaranteed or endorsed by the publisher.

## Supplementary material

Supplementary Figure 1 GO and KEGG analysis of differentially expressed fatty acid metabolism-linked genes. **(A)**The result of GO enrichment analysis of differentially expressed fatty acid metabolism-linked genes. **(B)** The result of KEGG enrichment analysis of differentially expressed fatty acid metabolism-linked genes.

Supplementary Figure 2 Survival analysis and immune infiltration analysis of hub Genes. **(A, C, E)** Survival analysis of hub genes. **(B, D, F)** Immune infiltration analysis of hub genes.

Supplementary Figure 3 The relationship of gene expression and clinicopathological features, consisting of age, T stage, gender, clinical stage, N stage, grade, and M stage. **(A–G)** COL1A2. **(H–N)** COL3A1. **(O–U)** SPP1.
