# Supplementary figures and images for: Evaluation of aliphatic acid metabolism in bladder cancer with the goal of guiding therapeutic treatment
Source: Front Oncol. 2022 Aug 18;12:930038. doi: 10.3389/fonc.2022.930038 (PMC9433665; doi:10.3389/fonc.2022.930038)

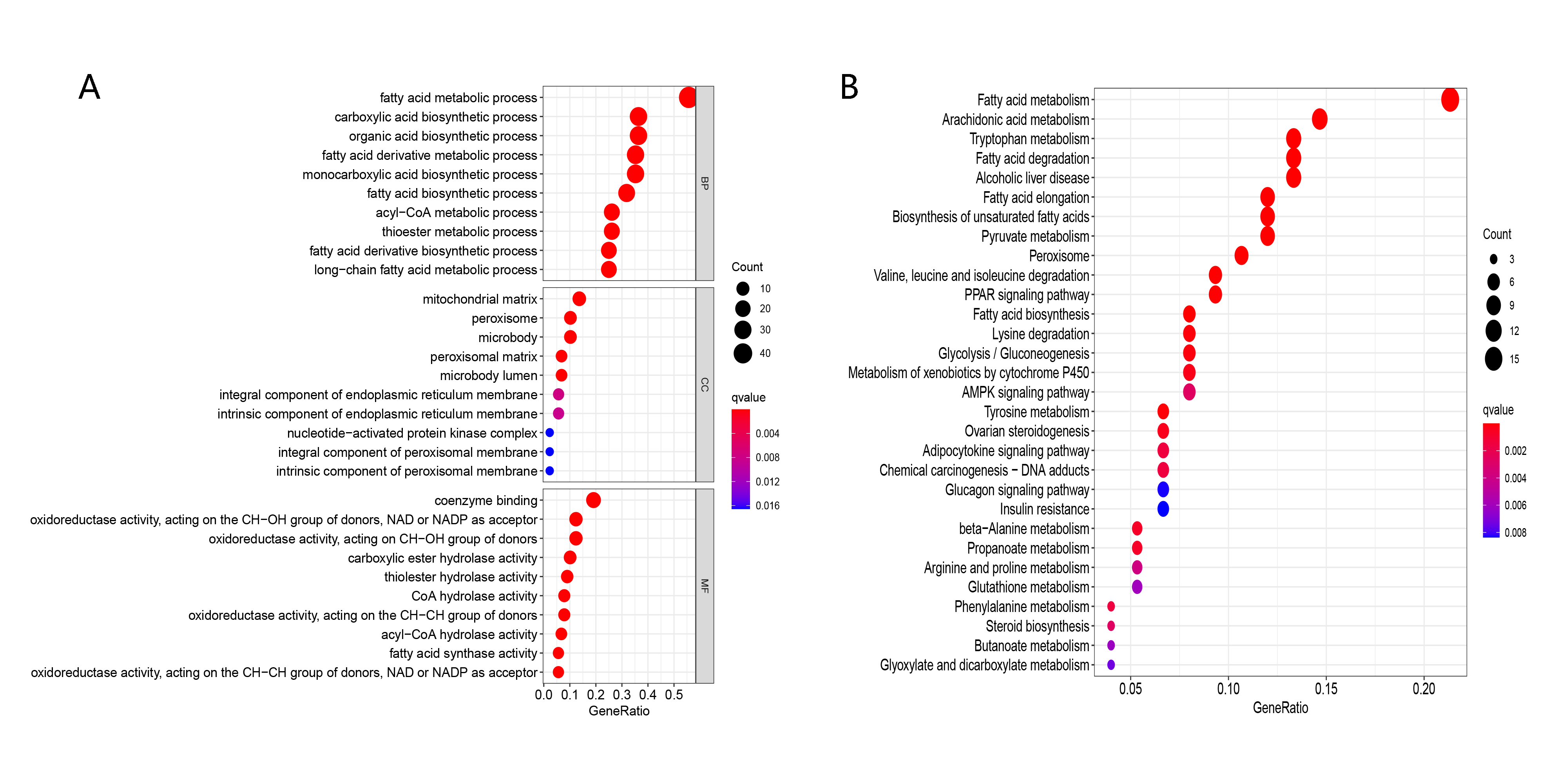

Supplement: Supplementary Figure 1 — GO and KEGG analysis of differentially expressed fatty acid metabolism-linked genes. (A)The result of GO enrichment analysis of differentially expressed fatty acid metabolism-linked genes. (B) The result of KEGG enrichment analysis of differentially expressed fatty acid metabolism-linked genes. [file Image_1.tif]

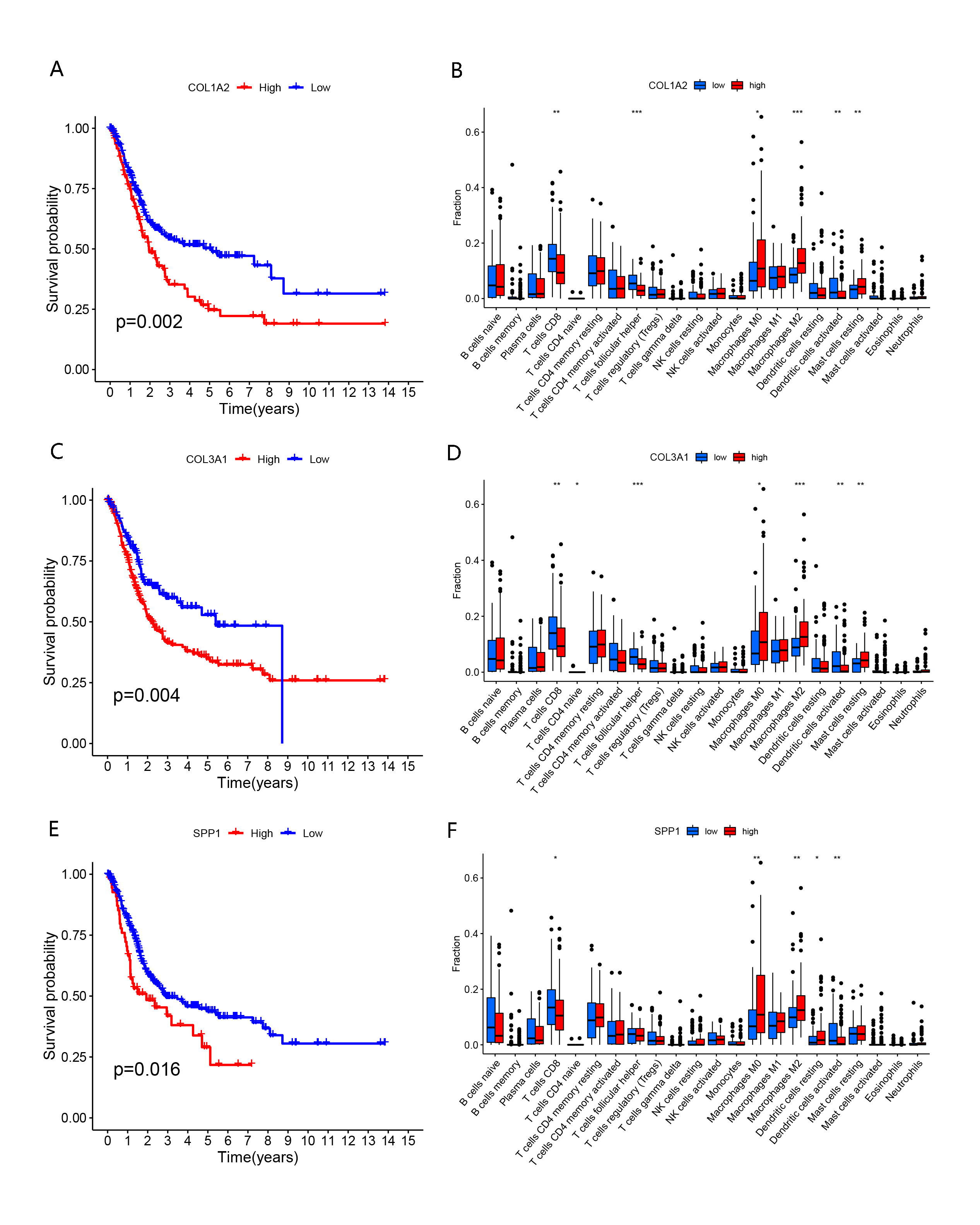

Supplement: Supplementary Figure 2 — Survival analysis and immune infiltration analysis of hub Genes. (A, C, E) Survival analysis of hub genes. (B, D, F) Immune infiltration analysis of hub genes. [file Image_2.tif]

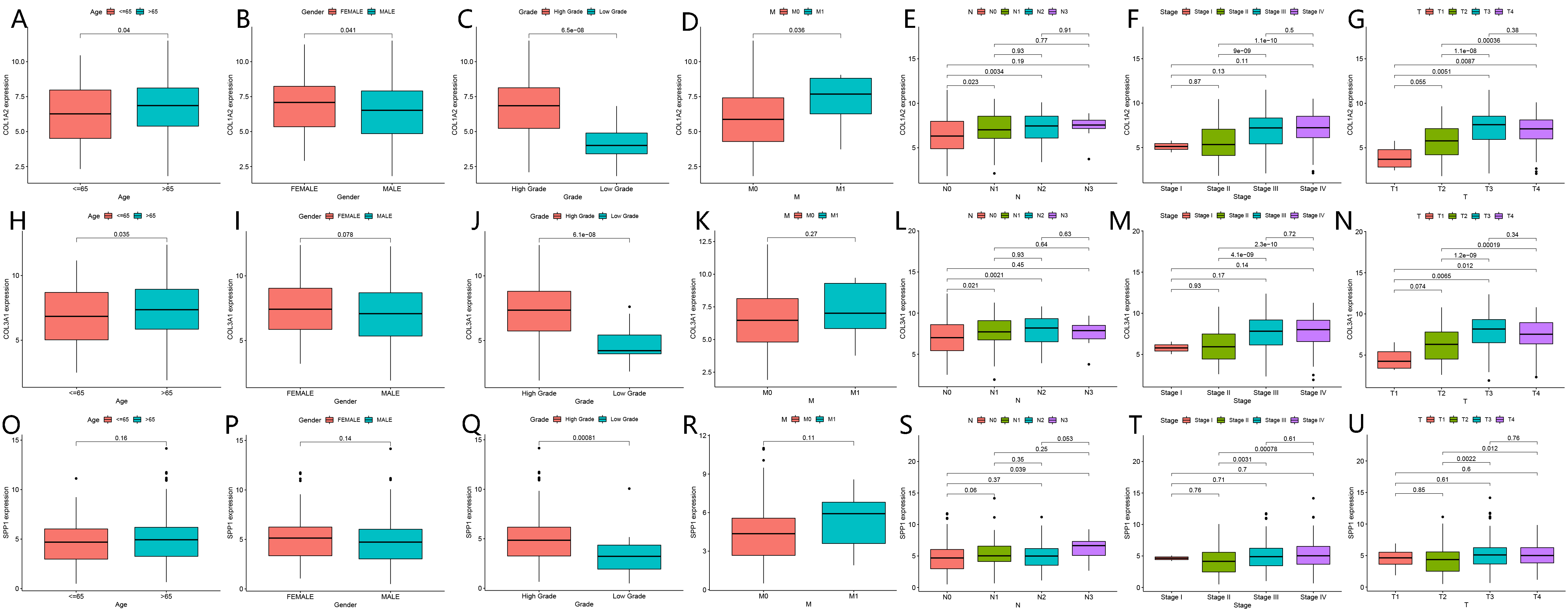

Supplement: Supplementary Figure 3 — The relationship of gene expression and clinicopathological features, consisting of age, T stage, gender, clinical stage, N stage, grade, and M stage. (A–G) COL1A2. (H–N) COL3A1. (O–U) SPP1. [file Image_3.tif]
